# Usefulness of Spleen Index and Alkaline Phosphatase Level for Predicting Post‐Liver Biopsy Bleeding

**DOI:** 10.1002/jgh3.70183

**Published:** 2025-05-20

**Authors:** Hirohito Takeuchi, Katsutoshi Sugimoto, Tatsuya Kakegawa, Hiroshi Takahashi, Takuya Wada, Masakazu Abe, Yu Yoshimasu, Kazuharu Harada, Masataka Taguri, Takao Itoi

**Affiliations:** ^1^ Department of Gastroenterology and Hepatology Tokyo Medical University Tokyo Japan; ^2^ Department of Health Data Science Tokyo Medical University Tokyo Japan

**Keywords:** ALP level, bleeding complications, liver biopsy, spleen index, splenomegaly

## Abstract

**Aims:**

The significance of liver biopsy is increasing with an increase in chronic liver disease and gene panel testing. Although non‐invasive methods such as elastography and biomarkers assess liver fibrosis, biopsy remains the definitive diagnostic gold standard. We evaluated the predictors of bleeding complications in liver biopsies.

**Methods and Results:**

A total of 697 patients were enrolled in this study between May 2017 and October 2022. We examined bleeding complications and procedures following liver biopsy and the liver biopsy needle size, blood test results, and spleen index to determine factors related to bleeding complications. Bleeding complications occurred in 23 patients (3.3%), including 20 cases at the liver puncture site, two instances of biliary bleeding, and one intercostal artery injury. The treatments varied and included hepatic arterial embolization (2 patients, 0.3%), blood transfusion therapy (3 patients, 0.4%), radiofrequency ablation (2 patients, 0.3%), endoscopic nasobiliary drainage (1 patient, 0.1%), and other treatments. In multivariate and ROC analyzes, a higher spleen index (Odds ratio: 1.13 [1.07–1.20], AUC: 0.74, optimal cut‐off value: 16.2, sensitivity: 0.74, specificity: 0.64) and ALP level (Odds ratio: 1.00 [1.00–1.01], AUC: 0.71, optimal cut‐off value: 94.5, sensitivity: 0.83, specificity: 0.49) were associated with an increased risk of bleeding. Other significant factors influencing bleeding included age, PT‐INR, needle size, and Child–Pugh score.

**Conclusions:**

The identified risk factors included spleen index and ALP level, particularly in relation to bleeding complications during liver biopsy. Therefore, these predictors should be considered before performing a liver biopsy.

## 1 Introduction

The significance of liver biopsy is increasing with the diagnosis of chronic liver disease and widespread use of gene panel testing [1, 2]. Although liver stiffness measurements, such as magnetic resonance and ultrasound (US) elastography, and non‐invasive biomarkers can be used to assess the degree of liver fibrosis, liver biopsy remains the gold standard for definitive diagnosis [3, 4, 5]. Liver biopsy is reportedly a safe procedure; however, bleeding complications occur at a low frequency, and deaths have been reported [6, 7, 8]. Therefore, particular attention should be directed toward preventing bleeding complications [9]. Several risk factors have been reported for bleeding complications in liver biopsy. Specifically, increased complication risk was associated with multiple puncture passes, platelet counts of < 50 000/μL, and prothrombin time‐international normalized ratio (PT‐INR) level of > 1.4 [10, 11]. Although guidelines for liver biopsy are available in the United Kingdom and France, none exist in Japan [2, 7].

We performed liver biopsies safely with a low frequency of bleeding complications. Additionally, we created a database of liver biopsies from May 2017 to investigate the complications of liver biopsy, including patient clinical information, blood test data, number of puncture passes, and biopsy needle gage. Therefore, this study aimed to investigate 697 liver biopsies performed at our hospital and examine the predictive factors of bleeding complications.

## 2 Materials and Methods

### 2.1 Patients and Methods

This study was approved by the Internal Review Board and Ethics Committee of Tokyo Medical University (Approval No.: T2020‐0323) and was conducted in accordance with the principles of the Declaration of Helsinki. The requirement for written informed consent was waived owing to the retrospective nature of the study. A total of 710 patients who underwent liver biopsy at our institution between May 2017 and October 2022 were enrolled in this study. Eleven patients who underwent splenectomy for whom the spleen index could not be measured, and two who underwent transvenous liver biopsy were excluded. Finally, 697 patients were included in the analysis. Bleeding complications and post‐liver biopsy procedures were also assessed. The liver biopsy needle size, number of puncture passes, blood test results, spleen index, and patient background were analyzed in 697 patients to determine the factors contributing to bleeding complications.

### 2.2 Liver Biopsy

Ten hepatologists with 11–23 years of liver biopsy experience and residents with 4–6 years of clinical experience performed liver biopsies under US guidance. Residents performed the liver biopsies under the supervision of at least two hepatologists. An Aplio‐i800 US system (Canon Medical Systems, Tochigi, Japan) was used. The patients were initially administered local anesthesia, and the specimens were collected while avoiding large blood vessels. We performed procedures to assess chronic liver disease (non‐targeted) and liver tumor biopsy (targeted). TruCore Needle (Argon Medical Devices Inc., Plano, Texas, USA) (16G and 18G) from May 2017 to July 2020. The CorVocet needle (Merit Medical Systems Inc., South Jordan, USA) (18G) was used after August 2020, and the 20G needle employed was a TOP aspiration biopsy needle (TOP Co., Tokyo, Japan). The 16G and 18G needles were mainly used to assess chronic liver disease, whereas liver tumor biopsy was performed using 18G and 20G needles. Additionally, a 20G needle was used as a control for liver tumors in some cases. All biopsy specimens were fixed in 10% formalin and embedded in paraffin. After tissue collection, US Doppler was used to confirm the absence of bleeding, and the Morrison fossa, gallbladder, and perihepatic area were observed. Patients receiving anti‐thrombotic therapy stopped taking their medications for a defined period to prevent bleeding and resumed them the day after the liver biopsy.

### 2.3 Database of Liver Biopsy

The entry of the liver biopsy database began in May 2017. The information entered included patient age, sex, clinical diagnosis, histopathological diagnosis, number of puncture passes, needle size, bleeding complications, other complications, treatment for complications, history of anti‐thrombotic medications, presence of platelet‐promoting drugs, presence of hemophilia, presence of portal hypertension, spleen index, presence of splenectomy, years of operator experience (hepatologist certification), blood tests (white blood cell count, platelet [PLT] count, hemoglobin, PT‐INR, albumin [Alb], aspartate aminotransferase [AST], alanine aminotransferase [ALT], γ‐glutamyl transpeptidase [γ‐GTP], alkaline phosphatase [ALP] (International Federation of Clinical Chemistry), and total bilirubin [T‐Bil] levels), presence of encephalopathy and ascites, and Child–Pugh score and classification.

### 2.4 Spleen Index

**FIGURE 1:**
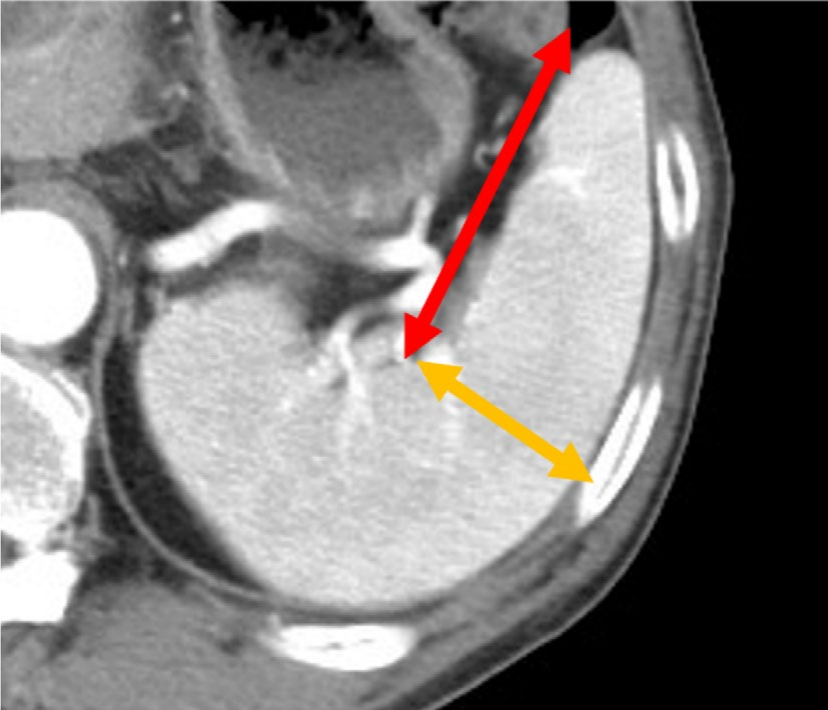
Measurement of spleen index. The spleen index was calculated using the formula established by the First Department of Internal Medicine at Chiba University. It was determined as the product of the diameter from the splenic hilum to the anterior margin of the spleen and the diameter perpendicular to this measurement. Splenomegaly was defined as a size of ≥ 20 cm^2^.

In this study, the spleen index was calculated using a formula established by the First Department of Internal Medicine at Chiba University. It was calculated as the product of the diameter from the splenic hilum to the anterior margin of the spleen and the diameter perpendicular to this measurement (Figure 1). Splenomegaly was defined as a size of ≥ 20 cm^2^. US, computed tomography (CT), and magnetic resonance imaging scans used for analysis were performed on the day of liver biopsy or within 3 months.

### 2.5 Bleeding Complications

Bleeding complications in liver biopsy were defined as intra‐abdominal and hepatic bleeding of grade ≥ 2 according to the Common Terminology Criteria for Adverse Events (CTCAE) version 5.0. Cases that required procedures such as transcatheter arterial embolization (TAE), radiofrequency ablation (RFA), endoscopic nasobiliary drainage (ENBD), blood transfusion therapy, fasting, and deferred discharge were classified as Grade 3. Cases in which intra‐abdominal bleeding was observed on ultrasound, CT, or other imaging studies and required only follow‐up were classified as Grade 2. Biliary bleeding and intercostal artery injuries resulting from liver biopsy were included.

### 2.6 Statistical Analysis

All statistical analyzes were performed using IBM SPSS software (version 28, IBM Corp., Armonk, NY, USA). Continuous variables were summarized using medians (interquartile ranges), and group comparisons were conducted using the Mann–Whitney *U* test. Categorical variables were summarized using counts and percentages, and group comparisons were performed using Fisher's exact test. Multiple logistic regression analysis was conducted to identify the significant predictors of bleeding complications. In addition to the full model, which included all candidate variables, a reduced model was constructed by removing irrelevant variables. A threshold of *p* > 0.157 was used to maximize the predictive performance of the model during variable reduction [12]. For the model's predicted probabilities and important factors, receiver operating characteristic (ROC) analysis was performed. The area under the curve (AUC), 95% confidence interval, optimal cut‐off values, sensitivity, specificity, positive predictive value, and negative predictive value were reported based on the Youden Index. Statistical significance was set at *p* < 0.05.

## 3 Results

### 3.1 Baseline Patient Characteristics

Table 1 summarizes the baseline patient characteristics. A total of 697 patients with a median age of 64 years (interquartile range, 48–74 years) were included in this study. Among them, 411 and 286 patients were male and female, respectively. Blood test results before liver biopsy showed a median PLT count, PT‐INR level, Alb level, AST level, ALT level, γ‐GTP level, ALP level, and T‐Bil level of 21.2 × 10^4^/μL, 1.00, 4.0 g/dL, 36 IU/L, 32 IU/L, 74 IU/L, 97 IU/L, and 0.65 mg/dL, respectively. Additionally, the distribution of Child–Pugh scores, spleen index, years of operator experience, and the number of puncture passes were 5 (5), 13.8 (10.2–19.3), 10 (8–11) years, and 2 (1, 2), respectively. No cases of hemophilia or the use of platelet‐promoting or anti‐thrombotic medications were observed among the bleeding cases.

**TABLE 1:** Baseline patient characteristics and factors predicting bleeding.

	All patients (*n* = 697)	Bleeding (−) (*n* = 674)	Bleeding (+) (*n* = 23)	*p*
Age, years	64 (48–74)	64 (48–74)	61 (52–76)	0.781*
Sex				0.535†
Male	411 (59%)	396 (96.3%)	15 (3.6%)	
Female	286 (41%)	278 (97.2%)	8 (2.8%)	
Blood test before liver biopsy				
Platelet counts (×10^4^/μL)	21.2 (15.3–27.8)	21.4 (15.6–28.0)	17.7 (12.0–21.6)	0.012*
PT‐INR	1.00 (0.95–1.07)	21.4 (15.6–28.0)	17.7 (12.0–21.6)	0.530*
Albumin (g/dL)	4.0 (3.6–4.3)	4.0 (3.6–4.3)	3.5 (3.1–4.1)	0.008*
AST (IU/L)	36 (25–59)	36 (24–58)	48 (31–92)	0.033*
ALT (IU/L)	32 (18–69)	31 (18–68)	35 (24–79)	0.123*
γ‐GTP (IU/L)	74 (37–169)	73 (37–167)	127 (57–447)	0.008*
ALP (IU/L)	97 (73–145)	97 (73–143)	147 (98–336)	< 0.001*
T‐Bil (mg/dL)	0.65 (0.48–0.90)	0.65 (0.48–0.89)	0.78 (0.57–1.06)	0.132*
Ascites				0.399^†^
Ascites (+)	15 (2.2%)	14 (93.3%)	1 (6.7%)	
Ascites (−)	682 (97.8%)	660 (96.8%)	22 (3.2%)	
Child–Pugh score	5 (5–5)	5 (5–5)	6 (5–7)	< 0.001*
Spleen index (cm^2^)	13.8 (10.2–19.3)	13.7 (10.1–18.9)	21.6 (14.4–33.0)	< 0.001*
Needle size				0.013^†^
16G	148 (21.2%)	141 (95.3%)	7 (4.7%)	
18G	321 (46.1%)	306 (95.3%)	15 (4.7%)	
20G	228 (32.7%)	227 (99.6%)	1 (0.04%)	
Years of operator experience	10 (8–11)	10 (8–11)	10 (8–14)	0.812*
Number of passes	2 (1–2)	2 (1–2)	1 (1–2)	0.571*
Anti‐thrombotic medications				0.175^†^
Medication (+)	50 (7.2%)	0 (0%)	50 (100%)	
Medication (−)	647 (92.8%)	624 (96.4%)	23 (3.6%)	

*Note:* The continuous variables are summarized as median (interquartile range), and the categorical variables are summarized as count (percent). *P*‐values for the continuous variables are based on Mann–Whitney *U* test*, and those for the categorical variables are based on Fisher's exact test^†^.

Abbreviations: ALT, alanine aminotransferase; ALP, alkaline phosphatase; AST, aspartate aminotransferase; γ‐GTP, γ‐glutamyl transpeptidase; PT‐INR, prothrombin time international normalized ratio; T‐Bil, total bilirubin.

### 3.2 Predictive Factors for Bleeding Complications

**FIGURE 2:**
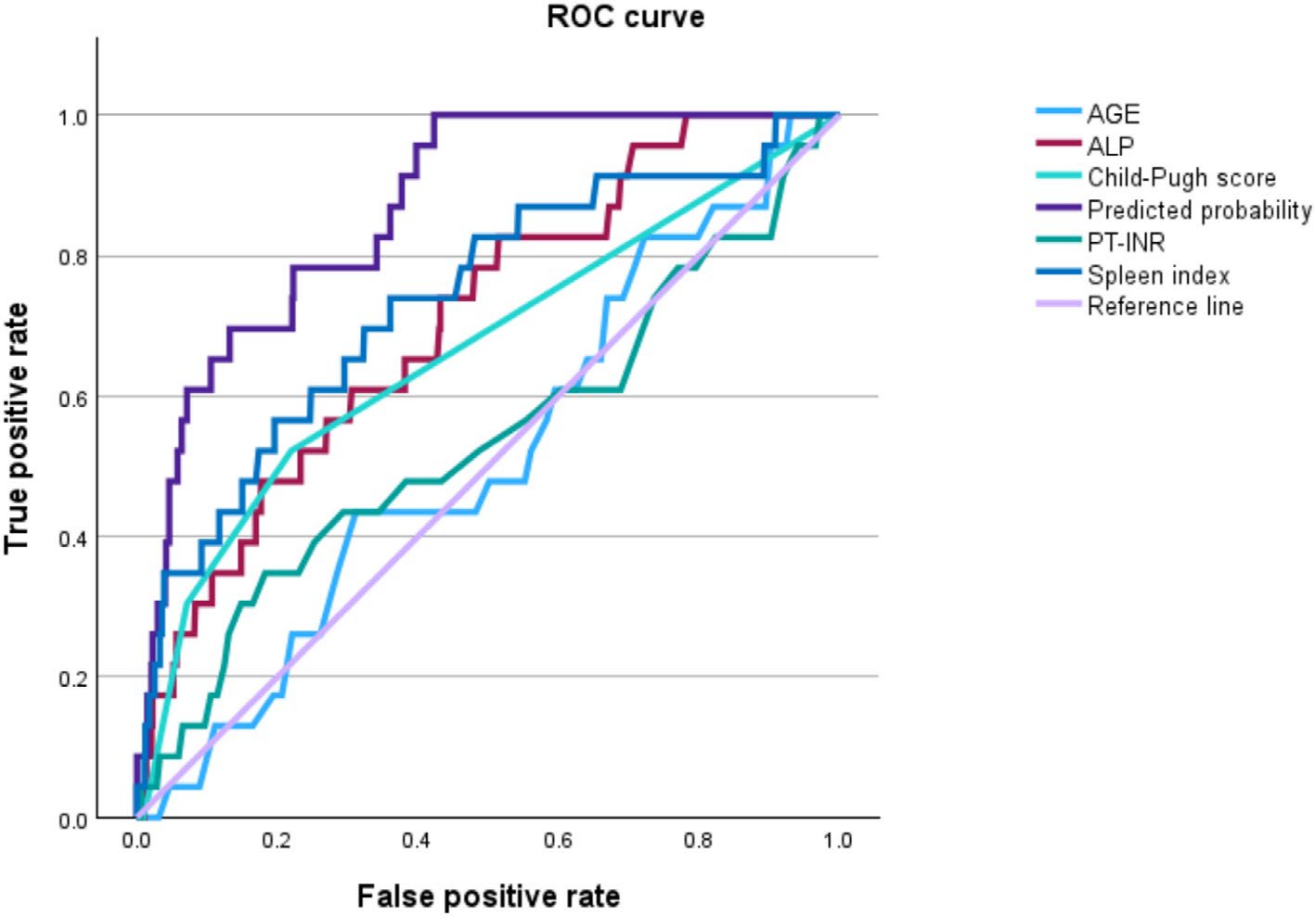
ROC analysis of predictors of bleeding in liver biopsy. Receiver operating characteristic (ROC) analysis results. The area under the ROC curve (AUC), optimal cut‐off value, sensitivity, and specificity are described in Table 3. ALP, alkaline phosphatase, PT‐INR, prothrombin time international normalized ratio.

Table 1 presents the factors that predicted bleeding complications. Among the 697 eligible patients, 23 experienced bleeding, whereas 674 did not. Significant differences were found between the bleeding and non‐bleeding groups in PLT count (*p* = 0.012), Alb level (*p* = 0.008), AST level (*p* = 0.033), γ‐GTP level (p = 0.008), ALP level (*p* < 0.001), Child–Pugh score (*p* < 0.001), spleen index (p < 0.001), and needle size (*p* = 0.013). Table 2 displays the results of the multiple logistic regression analysis. In the full model, the spleen index was significant (*p* < 0.001) with an odds ratio of 1.13 (1.07–1.20). ALP was not significant (*p* = 0.28), with an odds ratio of 1.00 (1.00–1.01). The 20G needle was significant (*p* = 0.049), with an odds ratio of 0.11 (0.01–0.99). The reduced model included age, PT‐INR, ALP, Child–Pugh score, spleen index, and needle size (16G vs. 18G and 16G vs. 20G). The odds ratios for these variables were 1.04, 0.01, 1.00, 1.60, 1.13, 1.07, and 0.09, respectively. Concerns regarding multicollinearity were raised due to relatively high Pearson's correlation coefficients observed between certain variable pairs, such as AST and ALT (*r* = 0.717) and γ‐GTP and ALP (r = 0.692). Consequently, a sensitivity analysis was performed by excluding each of these variables from the model one by one. In the model excluding γ‐GTP, ALP was significantly associated with bleeding complications (OR = 1.003 [1.001–1.005], *p* = 0.014), and in the model excluding ALP, γ‐GTP was significantly associated (OR = 1.002 [1.000–1.004], *p* = 0.015). There was no notable influence on the model when AST and ALT were excluded. Figure 2 presents ROC curves for bleeding complications, comparing the predictive performance of the model‐based probability and other key factors. Table 3 presents the AUC of the ROC curve, 95% confidence interval, *p*‐value, optimal cut‐off values, sensitivity, specificity, positive predictive value, and negative predictive value. The AUC for the spleen index and ALP was 0.74 (optimal cut‐off value = 16.2; sensitivity = 0.74; specificity = 0.64) and 0.71 (optimal cut‐off value = 94.5; sensitivity = 0.83; specificity = 0.49), respectively.

**TABLE 2:** Multivariate analysis of factors predicting bleeding.

	Full model			Reduced model
	Odds ratio	95% CI	*p*	Odds ratio
Age, years	1.02	(0.99–1.06)	0.23	1.04
Sex, Male Female	0.91 (ref)	(0.33–2.54)	0.86	
Platelet counts	0.97	(0.92–1.03)	0.30	
PT‐INR	0.02	(0–3.10)	0.13	0.01
Albumin	0.88	(0.18–4.33)	0.88	
AST	1.00	(0.98–1.01)	0.55	
ALT	1.00	(0.99–1.01)	0.55	
γ‐GTP	1.00	(1.00–1.00)	0.39	
ALP	1.00	(1.00–1.01)	0.28	1.00
T‐Bil	0.46	(0.18–1.17)	1.10	
Ascites	0.13	(0.01–2.89)	0.20	
Child–Pugh score	2.58	(0.74–9.00)	0.14	1.60
Spleen index	1.13	(1.07–1.20)	< 0.001	1.13
Needle size 16G	(ref)			
Needle size 18G	1.16	(0.36–3.68)	0.80	1.07
Needle size 20G	0.11	(0.01–0.99)	0.049	0.09
Years of operator experience	1.020	(0.92–1.14)	0.73	
Number of passes	0.92	(0.50–1.69)	0.79	

*Note:* The *p*‐value from the Wald test under the null hypothesis of odds ratio = 1 was presented.

Abbreviations: ALP, alkaline phosphatase; ALT, alanine aminotransferase; AST, aspartate aminotransferase; γ‐GTP, γ‐glutamyl transpeptidase; PT‐INR, prothrombin time international normalized ratio; T‐Bil, total bilirubin.

**TABLE 3:** The AUC for factors predicting bleeding.

Factor	AUC	95% Confidence interval	*p*	Optimal cut‐off value	Sensitivity	Specificity	Positive predictive value	Negative predictive value
Predicted probability	0.87	0.81–0.93	0.000	0.015	1.00	0.58	0.075	1.00
Age	0.52	0.40–0.63	0.771	71.5	0.43	0.68	0.045	0.97
PT‐INR	0.54	0.40–0.67	0.579	1.10	0.35	0.82	0.061	0.97
ALP	0.71	0.61–0.81	0.000	94.5	0.83	0.49	0.052	0.99
Child–Pugh score	0.66	0.54–0.79	0.011	5.5	0.52	0.78	0.075	0.98
Spleen index	0.74	0.63–0.85	0.000	16.2	0.74	0.64	0.065	0.99

*Note:* The *p*‐values were presented under the null hypothesis that each factor has no contribution to prediction.

Abbreviations: ALP, alkaline phosphatase; AUC, area under the ROC curve; ROC, receiver operating characteristic.

### 3.3 Adverse Events and Treatments

Table 4 presents all adverse events. Overall, 29 (4.2%) adverse events were reported, with bleeding being the most common, occurring in 23 (3.3%) cases, intraperitoneal bleeding in 20 (2.9%), biliary bleeding in 2 (0.3%), and intercostal artery injury in 1 (0.1%). After liver biopsy, three patients (0.4%) had abdominal pain and used analgesics, and three (0.4%) had a fever of > 38°C. No liver biopsy‐related deaths were observed.

**TABLE 4:** All adverse events (*n* = 697).

Adverse events	Bleeding (+)	Bleeding (−)	*n* (%)
Intraperitoneal bleeding	20	0	20 (2.9%)
Biliary bleeding	2	0	2 (0.3%)
Intercostal artery injury	1	0	1 (0.1%)
Fever (> 38.0°C)	0	3	3 (0.4%)
Pain	0	3	3 (0.4%)
Death	0	0	0 (0%)
Total	23 (3.3%)	6 (0.9%)	29 (4.2%)

### 3.4 Characteristics of Bleeding Cases and Treatments of Complications (*n* = 23)

Table 5 summarizes the characteristics of patients with bleeding observed in this study (*n* = 23). It presents a diverse range of conditions, including metabolic dysfunction‐associated steatohepatitis, autoimmune hepatitis, primary biliary cholangitis, graft‐versus‐host disease (GVHD), hepatocellular carcinoma, liver cirrhosis, and other diagnoses. The treatments administered included TAE, blood transfusion therapy, RFA, ENBD, fasting, and deferred discharge, with 2 (0.3%), 2 (0.3%), 1 (0.1%), 1 (0.1%), 1 (0.1%), and 1 (0.1%) patients, respectively. The CTCAE version 5.0 grade distribution for bleeding complications was as follows: Grade 1 (0), Grade 2 (15), Grade 3 (8), Grade 4 (0), and Grade 5 (0). Esophageal varices (6 cases) and portal hypertensive gastropathy (PHG) (2 cases) were commonly observed in patients requiring treatment.

**TABLE 5:** Characteristics of bleeding cases (*n* = 23).

	Bleeding cases (*n* = 23)
Pathological diagnosis	HCC 8, MASH 3, MASH‐LC 2, GVHD 2, AIH 1, PBC 1, PBC + AIH 1, Lymphoma 1, Hemosiderosis 1, Meta of lung cancer 1, Meta of esophageal cancer 1, Meta of sebaceous adenocarcinoma 1
Treatment of complication	Observation 15 (65%), TAE and blood transfusion therapy 2 (8.7%), RFA 2 (8.7%), ENBD 1 (4.3%), Blood transfusion therapy only 1 (4.3%), Fasting 1 (4.3%), Discharge postponement 1 (4.3%)
CTCAE version 5.0 (Grade 1/2/3/4/5)	0/15/8/0/0
Portal hypertension (+) (%)	8 (35%)
	Esophageal varix 6 (26%), PHG 2 (8.7%)

Abbreviations: AIH, autoimmune hepatitis; CTCAE, common terminology criteria for adverse events; ENBD, endoscopic nasobiliary drainage; GVHD, graft‐versus‐host disease; HCC, hepatocellular carcinoma; LC, liver cirrhosis; MASH, metabolic dysfunction‐associated steatohepatitis; Meta, metastasis; PBC, primary biliary cholangitis; PHG, portal hypertensive gastropathy; RFA, radiofrequency ablation; TAE, transcatheter arterial embolization.

## 4 Discussion

This study evaluated the predictors of bleeding complications in the liver biopsies of 697 patients. Bleeding complications occurred in 3.3% (23/697) of the cases, with bleeding from the liver puncture site being the most common, followed by biliary bleeding and intercostal artery injury. Severe bleeding that required intervention occurred in 1.1% (8/697) of the cases, and the treatments included TAE, RFA, ENBD, and blood transfusion. Despite the small number of events, the spleen index and ALP level emerged as significant predictors of bleeding complications, even after adjusting for other factors in the multivariate analysis. Both spleen index and ALP level were associated with an increased risk of bleeding complications, a finding not previously reported and presented here for the first time. Furthermore, the spleen index can be measured using abdominal ultrasonography, and ALP levels can be easily determined using blood tests, making them important predictors that can be evaluated using less invasive methods. As shown in Table 5, patients with bleeding complications had a higher prevalence of portal hypertension‐related conditions, such as esophageal varices and PHG, which contributed to a higher spleen index. Previous reports have indicated a higher mortality rate in liver biopsies of patients with hematologic disorders, with treatment‐requiring bleeding complications observed in patients with conditions such as GVHD or lymphoma. In this study, 17% (4/23) of bleeding complications occurred in patients with hematologic disorders [13]. Regarding the liver biopsy needle size, the 20G needle group showed significantly fewer bleeding complications than the 16G and 18G groups, indicating that smaller‐gage needles were associated with fewer bleeding complications than larger‐gage needles.

Previous reports have indicated that the bleeding rate associated with liver biopsy ranges from 2% to 10% [9], with severe bleeding requiring intervention occurring in 0.1%–5% (0.5% [7], 0.6% [8], 0.1%–4.6% [9], 0.2% [14], 0.5% [15], 0.11% [16], 1.2% [17], 0.6% [18], 0.11% [19], 1.58% [20]) of cases. The incidence rate of bleeding complications from liver biopsies at our facility was 3.3% (23 cases), with severe bleeding requiring intervention in 1.1% of cases (8 cases). Although the criteria for the degree of hemorrhage vary according to previous reports, these rates are consistent with those reported in previous studies. Previous studies have shown that bleeding complications are associated with several risk factors. Specifically, a PLT count of < 50 000/μL is generally considered a risk factor, although some reports have suggested a risk even with a PLT count of < 60 000/μL [7, 10, 18]. In our study, PLT count did not show statistical significance in the multivariate analysis. Regarding coagulation abnormalities, a correlation was observed between prothrombin time and PT‐INR, with PT‐INR levels ranging from 1.3 to ≥ 1.4 considered a risk factor [1, 7, 9, 13, 14, 18, 19, 20]. In our study, PT‐INR also showed statistical significance in the multivariate analysis. In a report related to the coagulation system, fibrinogen levels < 60 and > 200 mg/dL and an activated partial thromboplastin time (APTT) exceeding 100 s were considered risk factors [19, 21]. In our study, we did not measure fibrinogen or APTT in most cases; therefore, they were excluded from the analysis. Reports have suggested that the use of anti‐thrombotic agents increases the risk of bleeding complications, whereas oral aspirin intake is not associated with bleeding [9, 19]. In our study, the use of anti‐thrombotic agents was temporarily discontinued in all patients in accordance with the surgical guidelines before liver biopsy. Furthermore, none of the 23 bleeding cases involved patients actively taking anti‐thrombotic agents, suggesting that appropriate discontinuation may mitigate bleeding risk. Reports have suggested a correlation between the number of puncture passes during biopsy and bleeding risk, whereas others have indicated no association [9, 10, 14]. In our study, no significant difference was found in the number of puncture passes between the bleeding and non‐bleeding groups. Several reports have also indicated that operator experience was not associated with bleeding risk, and no statistical significance was found for operator years of experience in this study [9, 14, 18, 22]. Previous reports on liver biopsy complications are limited. Age, platelet count, number of passes, and needle size may or may not be risk factors for bleeding, depending on the study [23, 24, 25]. Factors such as the low incidence of bleeding complications (0.1%–5%), patient characteristics, targeted versus non‐targeted biopsy, and operator skill may influence the results, leading to conflicting findings. We believe that it is important to maintain a detailed database and accumulate more cases. In our study, liver biopsy‐related bleeding was significantly associated with age, PT‐INR, ALP, Child–Pugh score, spleen index, and needle size. These factors are related to liver dysfunction or reserve capacity and may reflect coagulation abnormalities or portal hypertension [26].

In this study, 35% (8/23) of patients with bleeding complications were associated with esophageal varices or PHG. Furthermore, since the spleen index was identified as a bleeding risk factor in multiple logistic regression analysis, and bleeding occurred in 74% (17/23) of cases exceeding the optimal cut‐off value (16.2 cm^2^), portal hypertension with splenomegaly was considered a predictor of bleeding in liver biopsy. Spleen volume and index provide crucial information during the evaluation of esophageal varices and portal hypertension [27, 28, 29, 30]. Splenomegaly is caused by portal congestion, tissue hyperplasia, and fibrosis. Increased spleen size has been shown to lead to increased blood flow, potentially causing portal hypertension [31]. Heo et al. reported that the presence of splenomegaly and gastroesophageal varices on CT is useful in determining clinically significant portal hypertension (CSPH) in patients with chronic liver disease [32]. However, measuring spleen volume using CT imaging is cumbersome and raises concerns regarding radiation exposure and healthcare costs. Therefore, the spleen index plays a significant role. The spleen index is calculated as the product of the diameter from the splenic hilum to the anterior margin of the spleen and the diameter perpendicular to this measurement, providing a convenient technique for assessing the spleen status. The spleen index is useful because it can be easily measured using abdominal ultrasonography. We calculated the spleen index from the images captured during liver biopsy or close to that time point to evaluate the potential for portal hypertension. Liver biopsy should be performed cautiously to avoid inadvertent puncture of the blood vessels or bile ducts. A risk of difficulty arises in hemostasis, particularly in cases with a high spleen index, due to the high intravascular resistance when the liver biopsy needle punctures the portal vein. Splenomegaly in chronic liver disease presents with portal hypertension; however, it is unclear whether portal pressure is elevated in splenomegaly due to hematologic disease. Some lymphomas have been reported to cause sinistral portal hypertension (SPH), but the mechanism of bleeding in hematologic diseases with splenomegaly may differ [33]. This point requires further pathological investigation as more cases are accumulated. The results of the ROC analysis showed that a spleen index > 16.2 cm^2^ is associated with a risk of portal hypertension and may be linked to a higher risk of bleeding during liver biopsy.

In this study, ALP levels were significantly different in the multivariate analysis. Among the bleeding cases, 83% (19/23) were found to have exceeded the optimal cut‐off value for ALP (94.5 U/L). As shown in the Results section, there was also a strong correlation between ALP and γ‐GTP levels, suggesting that γ‐GTP may be a risk factor for bleeding during liver biopsy. We examined the association between ALP and bleeding because ALP was included in the reduced model. ALP is classified into four isozymes based on the site of expression: intestinal, placental, germline, and tissue‐nonspecific alkaline phosphatase (liver/bone/kidney [L/B/K] type), although their physiological functions are not yet fully understood. A high ALP level is known to indicate bile duct obstruction and hepatocellular damage [34]. Of the patients with elevated ALP (ALP > 94.5 U/L) and bleeding, 10 patients had targeted biopsies, with a histopathologic diagnosis of hepatocellular carcinoma (HCC) or metastatic liver cancer. The median tumor diameter was 8.1 cm (7.0–12.7 cm), and all were hypervascular tumors. Since ALP is not a biomarker for HCC, elevated ALP levels may be related to the presence of chronic liver disease or a hypervascular tumor with a high proliferative capacity that compresses the surrounding small bile ducts [35]. Furthermore, portal vein obstruction caused by bile duct compression and obstruction may contribute to hemorrhage due to secondary elevated portal pressure and increased intravascular pressure in the surrounding vessels [36, 37]. In contrast, nine patients had non‐targeted biopsies and had chronic liver and hematologic diseases. GVHD is characterized by small intrahepatic bile duct obstruction, biliary stasis, and lymphocytic infiltration into the portal area, which may result in elevated ALP levels and secondary portal venous pressure elevation [38]. These results suggest that the risk of bleeding from liver biopsy may be higher in patients with elevated ALP, hypervascular tumors that compress the surrounding bile ducts, chronic liver disease, and hematologic disorders such as GVHD.

There were two cases with a spleen index < 16.2 cm^2^ and ALP < 160 U/L. One was considered a technical failure due to intercostal artery injury, while the other involved liver metastasis from sebaceous adenocarcinoma. The specimen was very fragile, and the specimen volume was small; therefore, the number of passes was increased to more than four, which was considered the cause of bleeding.

In the comparison of liver biopsy needle sizes, Table 1 shows a significant difference between the bleeding and non‐bleeding groups (*p* = 0.013). Multivariate analysis (Table 2) revealed no significant difference between the 16G and 18G groups; however, a significant difference was observed between the 16G and 20G groups (*p* = 0.049). While there was no statistical difference in bleeding complications between the 18G and 16G needles, the 20G needle was significantly less prone to bleeding complications than the 16G needle. Previous reports have indicated no difference in bleeding complications between the 16G and 18G groups, a finding consistent with our study [9, 39, 40]. The bleeding volume and sample tissue content increased with needle diameter, which may explain why bleeding was particularly low with the 20G needle [41]. However, liver biopsies using 16G or 18G needles are necessary when a relatively large amount of tissue is required, such as for gene panel testing, highlighting the importance of selecting an appropriate needle based on the intended purpose.

This study had a few limitations. The number of bleeding complications was low (23 cases), which may have been insufficient to obtain reliable estimates in the multivariate analysis. Moreover, it was impossible to evaluate the AUC of the reduced model using a separate dataset, which may have led to overfitting. However, because the model is relatively simple with a limited number of factors, we believe overfitting is not a major concern. Therefore, it is necessary to continually increase the number of cases in future studies to investigate this aspect further. Although the procedures were performed under the supervision of hepatologists, differences in experience and technical skills among the operators may have occurred. We assumed that the liver biopsies were performed at a uniform level of technical proficiency, as we could not review all tests and videos in this study. Bleeding during the liver biopsy is typically categorized as a hepatic artery, hepatic vein, or portal vein bleeding. However, determining the exact cause with respect to the type of vessel was difficult because of the retrospective nature of the study, and pulsed Doppler was not confirmed in all cases. Therefore, we aim to accumulate more cases in the future to rigorously confirm our results.

In conclusion, a spleen index > 16.2 cm^2^ and ALP > 94.5 U/L were useful in predicting bleeding during liver biopsy. When performing a liver biopsy, it is important to check these indicators to assess the indications and prepare for potential bleeding complications.

## Ethics Statement

This study protocol was approved by the Internal Review Board and Ethics Committee of Tokyo Medical University (Approval Number: T2020‐0323) and was conducted in accordance with the principles of the Declaration of Helsinki.

## Consent

The requirement for written informed consent was waived due to the retrospective nature of this study.

## Conflicts of Interest

The authors declare no conflicts of interest.

## References

[1] A. Khalifa and D. C. Rockey , “The Utility of Liver Biopsy in 2020,” Current Opinion in Gastroenterology 36 (2020): 184–191. 32097176 10.1097/MOG.0000000000000621 PMC10874678

[2] J. Neuberger , J. Patel , H. Caldwell , et al., “Guidelines on the Use of Liver Biopsy in Clinical Practice From the British Society of Gastroenterology, the Royal College of Radiologists and the Royal College of Pathology,” Gut 69 (2020): 1382–1403. 32467090 10.1136/gutjnl-2020-321299 PMC7398479

[3] A. Ozturk , M. C. Olson , A. E. Samir , and S. K. Venkatesh , “Liver Fibrosis Assessment: MR and US Elastography,” Abdominal Radiology 47, no. 9 (2022): 3037–3050. 34687329 10.1007/s00261-021-03269-4 PMC9033887

[4] H. Takeuchi , K. Sugimoto , H. Oshiro , et al., “Liver Fibrosis: Noninvasive Assessment Using Supersonic Shear Imaging and FIB4 Index in Patients With Non‐Alcoholic Fatty Liver Disease,” Journal of Medical Ultrasonics 2018, no. 45 (2001): 243–249. 10.1007/s10396-017-0840-3 29128938

[5] J. H. Zhou , J. J. Cai , Z. G. She , and H. L. Li , “Noninvasive Evaluation of Nonalcoholic Fatty Liver Disease: Current Evidence and Practice,” World Journal of Gastroenterology 25 (2019): 1307–1326. 30918425 10.3748/wjg.v25.i11.1307 PMC6429343

[6] G. Tian , D. Kong , T. Jiang , and L. Li , “Complications After Percutaneous Ultrasound‐Guided Liver Biopsy: A Systematic Review and Meta‐Analysis of a Population of More Than 12,000 Patients From 51 Cohort Studies,” Journal of Ultrasound in Medicine 39 (2020): 1355–1365. 31999005 10.1002/jum.15229

[7] J. B. Nousbaum , J. F. Cadranel , G. Bonnemaison , et al., “Clinical Practice Guidelines on the Use of Liver Biopsy,” Gastroentérologie Clinique et Biologique 26 (2002): 848–878. 12434096

[8] T. D. Atwell , R. L. Smith , G. K. Hesley , et al., “Incidence of Bleeding After 15,181 Percutaneous Biopsies and the Role of Aspirin,” AJR. American Journal of Roentgenology 194 (2010): 784–789. 20173160 10.2214/AJR.08.2122

[9] M. Midia , D. Odedra , A. Shuster , R. Midia , and J. Muir , “Predictors of Bleeding Complications Following Percutaneous Image‐Guided Liver Biopsy: A Scoping Review,” Diagnostic and Interventional Radiology 25 (2019): 71–80. 30644369 10.5152/dir.2018.17525 PMC6339629

[10] J. H. Boyum , T. D. Atwell , G. D. Schmit , et al., “Incidence and Risk Factors for Adverse Events Related to Image‐Guided Liver Biopsy,” Mayo Clinic Proceedings 91 (2016): 329–335. 26837481 10.1016/j.mayocp.2015.11.015

[11] H. Chi , B. E. Hansen , W. Y. Tang , et al., “Multiple Biopsy Passes and the Risk of Complications of Percutaneous Liver Biopsy,” European Journal of Gastroenterology & Hepatology 29 (2017): 36–41. 27556687 10.1097/MEG.0000000000000731

[12] G. Heinze , C. Wallisch , and D. Dunkler , “Variable Selection—A Review and Recommendations for the Practicing Statistician,” Biometrical Journal 60 (2018): 431–449. 29292533 10.1002/bimj.201700067 PMC5969114

[13] T. Ishikawa , E. Kodama , T. Kobayashi , et al., “Clinical Usefulness of Transjugular Liver Biopsy in Patients With Hematological Diseases With Liver Dysfunction,” Cureus 13 (2021): e19555, 10.7759/cureus.19555. 34917436 PMC8669626

[14] D. van der Poorten , A. Kwok , T. Lam , et al., “Twenty‐Year Audit of Percutaneous Liver Biopsy in a Major Australian Teaching Hospital,” Internal Medicine Journal 36 (2006): 692–699. 17040353 10.1111/j.1445-5994.2006.01216.x

[15] A. A. Sag , L. A. Brody , M. Maybody , et al., “Acute and Delayed Bleeding Requiring Embolization After Image‐Guided Liver Biopsy in Patients With Cancer,” Clinical Imaging 40 (2016): 535–540. 27133700 10.1016/j.clinimag.2015.11.004 PMC9383037

[16] D. C. Howlett , K. J. Drinkwater , D. Lawrence , S. Barter , and T. Nicholson , “Findings of the UK National Audit Evaluating Image‐Guided or Image‐Assisted Liver Biopsy. Part II. Minor and Major Complications and Procedure‐Related Mortality,” Radiology 266 (2013): 226–235. 23143026 10.1148/radiol.12120224

[17] N. Fotiadis , K. N. De Paepe , L. Bonne , et al., “Comparison of a Coaxial Versus Non‐Coaxial Liver Biopsy Technique in an Oncological Setting: Diagnostic Yield, Complications and Seeding Risk,” European Radiology 30 (2020): 6702–6708. 32666317 10.1007/s00330-020-07038-7 PMC7599171

[18] L. B. Seeff , G. T. Everson , T. R. Morgan , et al., “Complication Rate of Percutaneous Liver Biopsies Among Persons With Advanced Chronic Liver Disease in the HALT‐C Trial,” Clinical Gastroenterology and Hepatology 8 (2010): 877–883. 20362695 10.1016/j.cgh.2010.03.025 PMC3771318

[19] W. L. Chai , D. L. Lu , Z. X. Sun , et al., “Major Complications After Ultrasound‐Guided Liver Biopsy: An Annual Audit of a Chinese Tertiary‐Care Teaching Hospital,” World Journal of Gastrointestinal Surgery 15 (2023): 1388–1396. 37555112 10.4240/wjgs.v15.i7.1388 PMC10405124

[20] A. Maheux , Y. Purcell , S. Harguem , V. Vilgrain , and M. Ronot , “Targeted and Non‐Targeted Liver Biopsies Carry the Same Risk of Complication,” European Radiology 29 (2019): 5772–5783. 31076864 10.1007/s00330-019-06227-3

[21] A. Drolz , T. Horvatits , K. Roedl , et al., “Coagulation Parameters and Major Bleeding in Critically Ill Patients With Cirrhosis,” Hepatology 64 (2016): 556–568. 27124745 10.1002/hep.28628

[22] H. Takeuchi , K. Sugimoto , T. Kakegawa , et al., “The Usefulness of a Newly Developed Full‐Core Biopsy Needle in Liver Biopsy,” Hepatology Research 53 (2023): 247–257. 36355636 10.1111/hepr.13856

[23] W. Cao , Z. Cheng , L. Wang , X. Zhao , J. Li , and S. Zhou , “Analysis of Risk Factors of Bleeding Complications in Percutaneous Needle Biopsy of Liver Occupying Lesions,” International Journal of General Medicine 14 (2021): 2893–2899. 34234519 10.2147/IJGM.S313407 PMC8254094

[24] H. Jing , Z. Yi , E. He , et al., “Evaluation of Risk Factors for Bleeding After Ultrasound‐Guided Liver Biopsy,” International Journal of General Medicine 14 (2021): 5563–5571, 10.2147/IJGM.S328205. 34539186 PMC8444981

[25] T. A. Potretzke , L. J. Saling , W. D. Middleton , and K. A. Robinson , “Bleeding Complications After Percutaneous Liver Biopsy: Do Subcapsular Lesions Pose a Higher Risk?,” American Journal of Roentgenology 211 (2018): 204–210. 29708780 10.2214/AJR.17.18726

[26] S. Buob , A. N. Johnston , and C. R. Webster , “Portal Hypertension: Pathophysiology, Diagnosis, and Treatment,” Journal of Veterinary Internal Medicine 25 (2011): 169–186. 21382073 10.1111/j.1939-1676.2011.00691.x

[27] A. Borgheresi , R. Colleoni , M. Scalabrini , and D. Shigueoka , “The Splenic Index as Predictor of Bleeding and Variceal Recurrence in the Late Follow‐Up of Schistosomotic Patients After Exclusive Endoscopic Treatment,” ABCD. Arquivos Brasileiros de Cirurgia Digestiva 34, no. 4 (2022): e1638, 10.1590/0102-672020210002e1638. 35107500 PMC8846378

[28] E. Giannini , F. Botta , P. Borro , et al., “Platelet Count/Spleen Diameter Ratio: Proposal and Validation of a Non‐Invasive Parameter to Predict the Presence of Oesophageal Varices in Patients With Liver Cirrhosis,” Gut 52 (2003): 1200–1205. 12865282 10.1136/gut.52.8.1200 PMC1773759

[29] C. M. Lee , S. S. Lee , W. M. Choi , et al., “An Index Based on Deep Learning‐Measured Spleen Volume on CT for the Assessment of High‐Risk Varix in B‐Viral Compensated Cirrhosis,” European Radiology 31 (2021): 3355–3365. 33128186 10.1007/s00330-020-07430-3

[30] A. Berzigotti , S. Seijo , U. Arena , et al., “Elastography, Spleen Size, and Platelet Count Identify Portal Hypertension in Patients With Compensated Cirrhosis,” Gastroenterology 144 (2013): 102–111. 23058320 10.1053/j.gastro.2012.10.001

[31] M. Bolognesi , C. Merkel , D. Sacerdoti , V. Nava , and A. Gatta , “Role of Spleen Enlargement in Cirrhosis With Portal Hypertension,” Digestive and Liver Disease 34 (2002): 144–150. 11926560 10.1016/s1590-8658(02)80246-8

[32] S. Heo , S. S. Lee , S. H. Choi , et al., “CT Rule‐In and Rule‐Out Criteria for Clinically Significant Portal Hypertension in Chronic Liver Disease,” Radiology 309 (2023): e231208. 37906011 10.1148/radiol.231208

[33] X. H. Lv , L. L. Ma , W. Y. Fu , and Y. Xie , “Splenic Marginal Zone Lymphoma Leads to Sinistral Portal Hypertension,” Journal of Digestive Diseases 20 (2019): 45–48. 30549195 10.1111/1751-2980.12696

[34] U. Sharma , D. Pal , and R. Prasad , “Alkaline Phosphatase: An Overview,” Indian Journal of Clinical Biochemistry 29 (2014): 269–278. 24966474 10.1007/s12291-013-0408-y PMC4062654

[35] C. W. Huang , T. H. Wu , H. Y. Hsu , et al., “Reappraisal of the Role of Alkaline Phosphatase in Hepatocellular Carcinoma,” Journal of Personalized Medicine 12 (2022): 518. 35455635 10.3390/jpm12040518 PMC9030712

[36] P. Fickert , A. C. Lin , H. Ritschl , N. Hammer , and H. Denk , “Portal Venous Branches as an Anatomic Railroad for a Gut‐Bile Duct‐Axis,” Journal of Hepatology 79 (2023): e82–e84. 37044219 10.1016/j.jhep.2023.03.043

[37] J. S. Yu , K. W. Kim , M. S. Park , and S. W. Yoon , “Bile Duct Injuries Leading to Portal Vein Obliteration After Transcatheter Arterial Chemoembolization in the Liver: CT Findings and Initial Observations,” Radiology 221 (2001): 429–436. 11687687 10.1148/radiol.2212010339

[38] M. Salomao , K. Dorritie , M. Y. Mapara , and A. Sepulveda , “Histopathology of Graft‐Vs‐Host Disease of Gastrointestinal Tract and Liver: An Update,” American Journal of Clinical Pathology 145 (2016): 591–603. 27247365 10.1093/ajcp/aqw050

[39] F. Chen , B. Wang , and L. Z. Chen , “Comparison of Safety and Sensitivity in Diagnosis of Liver Lesion Between Ultrasound‐Guided 16 Gauges and 18 Gauges Core Needle Biopsy,” Zhonghua Yi Xue Za Zhi 87 (2007): 823–825. 17565865

[40] T. C. Hall , C. Deakin , G. S. Atwal , and R. K. Singh , “Adequacy of Percutaneous Non‐Targeted Liver Biopsy Under Real‐Time Ultrasound Guidance When Comparing the Biopince and Achieve Biopsy Needle,” British Journal of Radiology 90, no. 1080 (2017): 20170397, 10.1259/bjr.20170397. 28972801 PMC6047660

[41] D. M. Plecha , D. W. Goodwin , D. Y. Rowland , M. E. Varnes , and J. R. Haaga , “Liver Biopsy: Effects of Biopsy Needle Caliber on Bleeding and Tissue Recovery,” Radiology 204 (1997): 101–104. 9205229 10.1148/radiology.204.1.9205229

